# Case Report: Hemoptysis Caused by Pulmonary Tuberculosis Complicated With Bronchial Artery-Pulmonary Artery Fistula in Children

**DOI:** 10.3389/fped.2021.587342

**Published:** 2021-02-11

**Authors:** Huihui Zhu, Fangfang Lv, Ming Xu, Shunhang Wen, Yangming Zheng, Hailin Zhang

**Affiliations:** Department of Pediatric Pulmonology, The Second Affiliated Hospital & Yuying Children's Hospital, Wenzhou Medical University, Wenzhou, China

**Keywords:** children, hemoptysis, bronchial artery, pulmonary artery fistula, pulmonary tuberculosis

## Abstract

Bronchial artery-pulmonary artery fistula secondary to pulmonary tuberculosis is an important cause of hemoptysis in adults, but it's relatively rare in children. Bronchial artery-pulmonary artery fistulas are mostly congenital in children and may have no clinical manifestations in the early stage. Congenital bronchial artery-pulmonary fistula with pulmonary tuberculosis can lead to hemoptysis. From 2016 to 2020, two children with pulmonary tuberculosis complicated with bronchial artery and pulmonary artery fistula were admitted and treated in our hospital. We reminded pediatricians to pay attention to a variety of etiology combined with the possibility of children's hemoptysis.

## Introduction

Hemoptysis is one of the common symptoms of respiratory diseases in children. Massive hemoptysis can easily trigger asphyxia or shock and may be life-threatening. The causes of hemoptysis in children vary— respiratory issues or diseases such as bronchitis, pneumonia, pulmonary tuberculosis, presence of foreign bodies in the respiratory tract, and bronchiectasis can cause hemoptysis. Other rare potential causes include vascular malformations, congenital heart disease, idiopathic pulmonary hypertension, and pulmonary embolism (1). During clinical practice, we observed patients with multiple conditions causing hemoptysis. In this paper, we introduce two cases of hemoptysis caused by pulmonary tuberculosis complicated with bronchial artery-pulmonary artery fistula (BPF).

## Case Report

### Case 1

**Figure 1:**
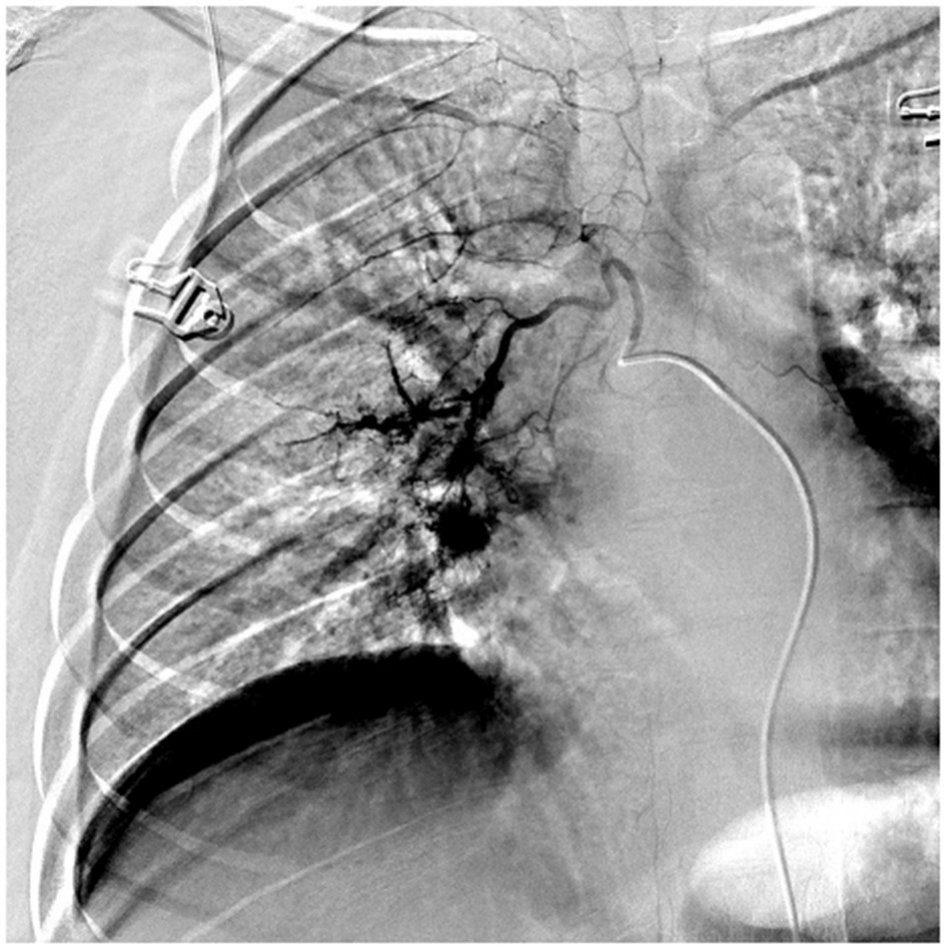
DSA showed that right bronchial artery was obviously thickened and tortuous and disordered, which showed bronchial arterio-pulmonary fistula.

A 15-year-old, previously healthy male, presented to our hospital with a cough and hemoptysis for 7 days; hemoptysis occurred 7–8 times every day. Although afebrile, the patient coughed 20–30 ml of blood (bright red) each time. The patient had no personal or family history of hemoptysis, recurrence of respiratory tract infection, or tuberculosis. The cough reduced in response to azithromycin treatment recommended previously in another hospital for *Mycoplasma pneumoniae* pneumonia (MPP), but hemoptysis persisted. Laboratory test findings were normal in terms of blood components, blood coagulation function, and immunoglobulin levels, and negative for antineutrophil cytoplasmic antibody and antinuclear antibody series, autoantibody series, sputum culture and MP antibodies. Mild exudative changes in the middle field of the right lung were observed in the chest X-ray. CT angiography (CTA) findings suggested dilation and enlargement of the right bronchial artery, with no obvious abnormality in the thoracic aorta and bilateral pulmonary arteries, while sputum and blood inhalation were noted in the dorsal segment of the lower lobe of the right lung. A pulmonary fistula was seen in the right bronchial arteries on bronchial arteriography with digital subtraction angiography (DSA) (Figure 1). Hemoptysis resolved after embolization with microspheres and coils, so BPF was diagnosed. The BCG scar of the child was negative and he tested positive for PPD (12 ×12 mm). The interferon gamma release assay (IGRAs) for tuberculosis infection was 234.3 pg/mL (normal value <14 pg/mL). Bronchoscopy revealed inflammatory changes in the bronchus. The patient's sputum tested positive for acid-fast bacilli during bronchial brush examination; these findings confirmed pulmonary tuberculosis. Hemoptysis did not recur after anti-tuberculosis treatment and embolization.

### Case 2

**Figure 2:**
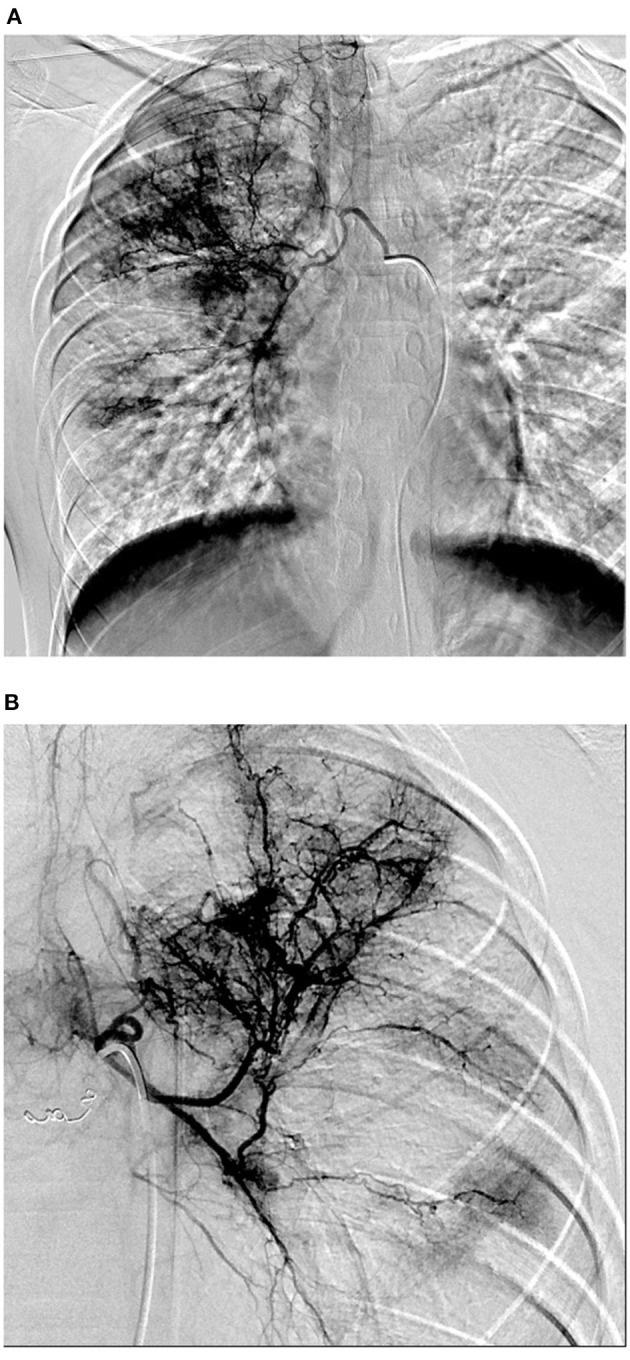
**(A,B)** DSA showed that bilateral bronchial arteries were obviously thickened and tortuous and disordered, which showed bronchial arterio-pulmonary fistula.

A 13-year-old male patient presented to our hospital with hemoptysis; the patient coughed 100 mL of bright red blood in 3 days. The patient experienced no chest pains, distress, sweating, pallor, or fever. CT demonstrated infectious lesions in both lungs (prone to tuberculosis and fungus infection). The patient denied a history of or contact with others with tuberculosis, wheezing, or rhinitis. The patient's family members indicated that they had no relevant history of tuberculosis. No other positive signs were found except for follicular hyperplasia and slight hyperemia in the posterior pharyngeal wall. Hemoptysis occurred several times after admission, with the patient coughing 20 mL of blood each time. Laboratory test findings were normal in terms of blood components, coagulation, and blood biochemistry, and negative for antibodies to atypical pneumonia. However, the patient tested positive for IGRAs with a value of 226.5 pg/mL (normal value <14 pg/mL) and his sputum was positive for acid-fast bacilli; PPD test findings were also positive (14 ×16 mm). Bronchoscopy revealed active bleeding in the bronchial lumen of the upper and lower lobes of the right lung. Exudation of both lungs and inhalation changes observed on CT suggested pulmonary hemorrhage. Bronchial artery angiography revealed obviously thickened, tortuous, and disordered bilateral bronchial arteries and a BPF (Figure 2). Therefore, the patient was diagnosed with BPF complicated with pulmonary tuberculosis. Hemoptysis did not recur after anti-tuberculosis treatment and embolization, as confirmed by regular follow up examinations.

## Discussion

Due to its hidden onset and atypical clinical symptoms, tuberculosis in children has not been sufficiently investigated. According to the Global Tuberculosis Report released in 2019 by WHO, around 10 million new cases were reported worldwide in 2018, 11% of which involved children younger than 15 years (1). China is one of the 30 countries with a high burden of tuberculosis, and the epidemic situation among children is not optimistic. The number of TB cases among children aged 0–14 years in China was ~100,000 in 2016, accounting for 11.17% of the total number of cases that year (2). With its generally slow onset, primary pulmonary tuberculosis is the most common type of TB infection in children. It might cause fever, cough, and wheezing in addition to symptoms of tuberculosis poisoning. Hemoptysis can occur in older children with caseous, invasive pulmonary tuberculosis or tracheobronchial tuberculosis (3). Therefore, the possibility of tuberculosis should be considered in cases of hemoptysis. Due to its lower sensitivity than CT, normal chest X-ray images can be seen at 25–40% of the pulmonary tuberculosis cases (4). Both cases discussed here presented with coughing, hemoptysis and abnormal CT results; moreover, chest pain was reported in Case 2. Despite denial of contact with confirmed TB cases and lack of typical symptoms of tuberculosis poisoning, we confirmed the diagnosis of pulmonary tuberculosis via a series of laboratory tests, comprising tests for PPD, IGRAs and anti-acid bacilli. Hence, the key to prevention and management of TB is to strengthen the awareness of diagnosis.

Massive hemoptysis incidence is rare in children with pulmonary tuberculosis; however, the amount of blood coughed during hemoptysis episodes was relatively large in both cases. For further examination, CTA and DSA exams were performed, indicating BPF in both cases. BPF can be a congenital systemic artery-pulmonary artery fistula, which is also known as congenital systemic artery-pulmonary artery malformation. Due to its characteristic of abnormal traffic between tortuous and dilated systemic arteries and pulmonary arteries, this rare congenital pulmonary disease is considered a pulmonary arteriovenous malformation (PAVM) (5). Nomenclatures of these lesions vary in the literature, and it is referred to as bronchial arteriovenous malformation, bronchopulmonary anastomosis, bronchopulmonary shunt, bronchial artery-pulmonary artery malformation, and fistulas, depending on the vessels involved (6). It has been reported that ~4% of all congenital PAVMs have a feeder artery arising from the systemic circulation, known as body arterial-pulmonary circulation fistula, which comprises two conditions; one is systemic artery-pulmonary artery fistula, and the other is pulmonary artery-pulmonary venous fistula with systemic arterial blood supply (7). The patients discussed here can be considered to have BPF as the bronchial artery functioned as the systemic feeder in both cases.

The cause of PAVMs remains unclear—the majority are congenital (80%), and a strong association with hereditary hemorrhagic telangiectasia (HHT) has been described in the literature (47–90%) (8). The acquisition has been associated with pathological factors such as cirrhosis, infectious diseases, trauma, mitral stenosis, Fanconi syndrome, and metastatic cancer (9). Some authors suggest that the traffic branches between pulmonary circulation and systemic circulation are occluded after birth when the baby breathes independently, and that BPF occurs when this occlusion fails. However, even if completely occluded, these traffic branches still hold the potential of reopening like a back door, and BPF might develop under specific conditions such as the presence of inflammation or tumor (10). Acquired processes like mental tension, cough, fatigue, and other factors can occasionally lead to same results due to the rapid pressure elevation in the lesion area, and hemoptysis occurs after the rupture of the bronchi artery or the bronchi artery-pulmonary artery anastomotic branch (11, 12). BPF is a pulmonary arteriovenous malformation, and although there might be no obvious clinical manifestations in some cases, the majority of patients present with hemoptysis (first symptom) accompanied by varying degrees of chest distress, shortness of breath, palpitation, and chest pain (13).

Infection, such as tuberculosis, can cause hemoptysis in cases with BPF; however, BPF is also an important pathologic mechanism for massive hemoptysis in patients with tuberculosis (14). Therefore, we attempted to understand whether tuberculosis triggered the occurrence of BPF in this case or whether these two pathological conditions coexisted independently. Thomas et al. (15) found a large area of caseous necrotizing granulomatosis during the histopathological examination of a 14-year-old girl with pulmonary arteriovenous fistula undergoing surgical excision, and speculated that the process of tuberculosis inflammation could recruit local and systemic arteries to supply blood to the inflammatory mass, which could help the tuberculosis foci absorb the local arteries, thus resulting in the fistula. Yoon et al. (16) reported three cases with recurrent massive hemoptysis as the main manifestation, which was caused by long-term pulmonary tuberculosis complicated with coronary artery causing bronchial arterial fistula. In a case reported by Saavedra et al. (13), systemic pulmonary arteriovenous fistula was observed in a 47-year-old male patient with a history of tuberculosis, suggesting a potential causal relationship between pulmonary tuberculosis and pulmonary arteriovenous fistula, and two possibilities were proposed. Firstly, the discovery of caseous necrotizing granuloma in the area of congenital vascular malformation may be an accidental phenomenon, since *Mycobacterium tuberculosis* affects the vascular area through a single preexisting fistula, in which case an additional fistula may develop. However, no extra fistula was noted in the patient's invasive angiographic study and CT scan. Moreover, no other common clinical features of Osler-Weber Rendu syndrome was observed in that case. Secondly, the possibility of systemic pulmonary arteriovenous fistula is related to the potential neovascularization process secondary to chronic inflammatory events of pulmonary tuberculosis and its sequelae. Inflammation may be a chronic angiogenic trigger for angiogenesis, erosion, and parietal lobe invasion. Pierce et al. (17) speculated that the destruction of pulmonary parenchyma, secondary to pulmonary tuberculosis, may lead to the accumulation of systemic arteries in this area, resulting in the formation of a fistula. Thus, it remains uncertain whether there is a causal relationship between tuberculosis and fistula formation. Whilst pulmonary tuberculosis may cause acquired bronchial artery-pulmonary artery fistula, it is a chronic inflammatory process, and in most adult patients, BPF develops years or even decades after the onset of tuberculosis (18). Thus, acquired BPF and pulmonary tuberculosis often occur several years apart. Moreover, BPF tends to occur in more severe lung lesions, such as cavity formation. If the patient has a short period of illness and imaging examinations do not indicate significant lung damage, the BPF is more likely to be congenital. The two patients discussed in this report were diagnosed with pulmonary tuberculosis for a short time, and no serious lung injuries were found following lung CT and bronchoscopy. Therefore, the diagnosis in two cases was congenital broncho-pulmonary fistulas. Unfortunately, no continuous angiography studies have been performed to objectively determine the natural history of BPF. It is well known that DSA is the gold standard for the diagnosis of BPF, while Qu et al. (18) found that DI-CTP scans have diagnostic value for detecting BPF in patients with tuberculosis and massive hemoptysis, providing an alternative diagnostic method. Hui et al. (19) reported 31 cases in which bronchial artery-pulmonary artery malformations were found during the interventional operation of patients with patent ductus arteriosus. Most of these patients were found to have small BPF during surgery, and some required no treatment. However, further investigation into this phenomenon and long-term follow-up to determine the possibility of occurrence, and possible triggers of hemoptysis and other symptoms in these children are warranted.

In summary, the timely detection of the cause of hemoptysis enabled prompt treatment of the patients. When hemoptysis occurs in children, we still need to consider the possibility of other rare causes, such as congenital tracheal artery-pulmonary fistula, after finding the common causes. CTA and DSA examinations may be required for the clarification of the underlying disease.

## Data Availability Statement

The original contributions presented in the study are included in the article/supplementary material, further inquiries can be directed to the corresponding author/s.

## Ethics Statement

Written informed consent was obtained from the individual(s) for the publication of any potentially identifiable images or data included in this article.

## Author Contributions

HZhu and FL collected patient data. HZhu, MX, SW, and YZ analyzed the data and wrote the manuscript. HZha reviewed and revised the manuscript. All authors contributed to the article and approved the submitted version.

## Conflict of Interest

The authors declare that the research was conducted in the absence of any commercial or financial relationships that could be construed as a potential conflict of interest.
